# Patient-reported outcome measures in hemodialysis patients: results of the first multicenter cross-sectional ePROMs study in France

**DOI:** 10.1186/s12882-021-02551-3

**Published:** 2021-10-30

**Authors:** Abdallah Guerraoui, Mathilde Prezelin-Reydit, Anne Kolko, Marie Lino-Daniel, Charlotte Dumas de Roque, Pablo Urena, Philippe Chauveau, Catherine Lasseur, Julie Haesebaert, Agnes Caillette-Beaudoin

**Affiliations:** 1 Calydial Dialysis Department, Calydial, CH Vienne Lucien Hussel, Lucien Hussel Hospital, Vienne, France; 2 AURAD-Aquitaine, Gradignan, France; 3 Association pour l’Utilisation du Rein Artificiel en région Parisienne (AURA) Paris, Paris, France; 4 Université Lyon, Université Claude Bernard Lyon 1, RESHAPE INSERM U1290, Lyon, France

**Keywords:** Chronic kidney disease, Fatigue, Hemodialysis, Quality of life, Patient reported outcome, PROMs

## Abstract

**Background:**

Kidney failure with replacement therapy and hemodialysis are associated with a decrease in quality of life (QOL). Self-reported QOL symptoms are not always prioritized by the medical team, potentially leading to conflicting priorities with patients. Electronic patient-reported outcome measures (ePROMs) allow physicians to better identify these symptoms. The objective was to describe the prevalence of symptoms self-reported by hemodialysis (HD) patients.

**Methods:**

A multicenter cross-sectional study was conducted in three HD centers. Patients were included if they were 18 years old or over treated with HD for at least 3 months in a center. Data were collected by the patient via a self-administered ePROMs questionnaire. Data included patient characteristics, post-dialysis fatigue and intensity, recovery time after a session, perceived stress, impaired sleep the day before the dialysis session, current state of health and the change from the past year. A multivariate analysis was conducted to identify relations between symptoms.

**Results:**

In total, we included 173 patients with a mean age of 66.2 years, a mean ± SD hemodialysis duration of 48.9 ± 58.02 months. The prevalence of fatigue was 72%. 66% had a high level of stress (level B or C). Recovery time was more than 6 h after a HD session for 25% of patients and 78% declared they had a better or unchanged health status than the previous year. Sleep disturbance was associated with cardiovascular comorbidities (OR 5.08 [95% CI, 1.56 to 16.59], *p* = 0.007).

**Conclusions:**

Fatigue and stress were the main symptoms reported by HD patients**.** The patient’s care teams should better consider these symptoms.

**Supplementary Information:**

The online version contains supplementary material available at 10.1186/s12882-021-02551-3.

## Background

Patients with chronic kidney disease (CKD) treated with hemodialysis (HD) require long-term care with a potentially negative impact on their quality of life (QoL) [1]. Patients treated with hemodialysis have a high symptomatic burden additionally impacting their QoL, which include pain, fatigue and stress [2].

These symptoms are, however, not always considered as a priority focus by the medical team caring for such patients and thus are not routinely collected despite their relevance to patients [3, 4]. The lack of physician focus on QoL may lead to conflicting medical and treatment priorities between a patient and their care team and potentially lead to undiagnosed and untreated symptoms. This is especially apparent for patients on maintenance dialysis [5–7].

The collection of electronic patient-reported outcome measures (ePROMs) is an innovative method to better take consider these symptoms [8]. Moreover, ePROMs allow patients to report their symptoms on a regular basis and allow the medical team to adapt treatment plans accordingly [8]. However, the use of ePROMs for CKD patients treated with HD in routine care remains limited [9, 10]. The objective of the study was to describe the prevalence of symptoms reported by patients treated with hemodialysis using ePROMs. The secondary objective was to explore predictive factors of the presence of the patient reported symptoms.

## Methods

### Study design

A multicenter cross-sectional observational study was conducted in three hemodialysis centers in France (Vienne, Bordeaux and Paris) between January and March 2020. Vienne regularly followed 90 patients, Bordeaux 128 and Paris 149. Patients were included if they were aged at least 18 years old and treated with hemodialysis for at least 3 months in one of the participating centers. Patients were excluded if they refused to participate in the study or could not read or understand French well enough to complete the questionnaires.

### Choice of symptoms

We chose to report the symptoms which were the most frequently reported in a preliminary qualitative study. This qualitative study was conducted to identify relevant symptoms reported by patients and affecting quality of life between dialysis sessions. A total of 20 patients were interviewed by a nephrologist trained to conduct semi-structured interviews. Patients were encouraged to provide examples and expand their answers to collect further details. Interviews were then transcribed and coded with thematic analysis to identify theme and subthemes from the data. Interviews were ceased when no new codes were identified and data saturation was reached [11]. We explored inter-dialysis symptoms and selected the three most frequent ones that were fatigue, stress-related symptoms and sleep disorders (Table 1). The interview guide, patient details and verbatim are provided in Additional file 1.

**Table 1:** Themes and subthemes identified during the preliminary qualitative phase with for patients with chronic kidney disease (CKD) treated with hemodialysis in the ePROMs study

Themes	Subthemes
Fatigue	Feeling tired
	Lack of energy
Mental symptoms	Feeling anxious
	Feeling irritable
	Feeling sad
	Feeling nervous
	Concern
Sleep disorders	Difficulty falling asleep
	Difficulty staying asleep
Other symptoms	Decreased appetite
	Decreased sexual desire
	Dry mouth
	Cramps
	Itching

### Data collection

ePROMs data were collected at one time with a self-administered by the patient ePROMs through an electronic tablet during a HD session or consultation by the patient or with the help of a caregiver if necessary. The collection of data through a tablet was chosen due to the ease of use and simple interface for the patients. The validation of the questionnaire required the questionnaire to be completed therefore there could not be any missing data.

Data collected included the presence of post-dialysis fatigue with a binary question (yes/no), its perceived intensity with a visual analog scale (VAS) ranged from 0 to 10 (0 was no perceived intensity and 10 was the most possible perceived intensity) [12], and the recovery time of this symptom after a session as a Likert scale question [13] (Table 2). Perceived stress (PSS 10) was evaluated using a scale adapted from Cohen and Williamson [14, 15] (Table 2). Sleep quality the day before the dialysis session was evaluated in the form of a Likert scale format, the current state of health of the patient using a VAS [16, 17], and the one-year change using a Likert scale were also collected. Patient characteristics were described from the patient’s medical records (demographics, dialysis situation, BMI, comorbidities, hemoglobin). Ethnicity was not collected in compliance with national regulations.

**Table 2:** Symptom scales chosen for patients with chronic kidney disease treated with hemodialysis in the ePROMs study

Symptoms	Indicator	Scale
Fatigue	Prevalence	Binary question: Yes/No
	Intensity	Visual analog scale from 0 (not tired) to 10 (very tired)
	Recovery time	Likert scale (less than 2 h, 2 to 6 h, 7 to 12 h, more than 12 h)
Stress	Intensity	Visual analog scale from 0 (not stressed) to 10 (very stressed)
	Severity	Perceived Stress Scale adapted from Cohen and Williamson A (<21): knows how to manage stress and adapt to find solutions B (21 to 26): knows to manage stress most of the time but it is not possible to manage stress in some situations. It is possible to learn stress management techniques C (≥27): does not know how to manage stress and perception of continuous threat which can negatively impact life and disease course.
Sleep disturbance	Sleep quality the night before dialysis compared to other nights	Likert scale (My sleep is better, my sleep is more or less good, my sleep is unchanged, my sleep is altered)
Overall health status	Current status	Visual analog scale from 0 (very poor health) to 10 (very good health)
	Comparison to 1 year before	Likert scale (poorer status, unchanged, more or less improved, improved)

### Statistical analysis

We included all patients with eligibility criteria during the study period, our aim was to include all patients during a 3 month period to be representative of patients. Continuous variables were described using means ± standard deviations (SD) or medians and interquartile ranges, according to the normality of their distribution. Numbers and percentages were used for qualitative variables. We first described the prevalence of each ePROM with an estimated 95% confidence interval [95% CI]. We compared patient characteristics and ePROMS between the three participating centers. Association between ePROMs (recovery time of fatigue (≤6-h versus> 6-h), perceived stress scale (PSS) stress level (A versus BC), sleep quality (disrupted if answer was “altered” versus not disrupted) and patients’ characteristics and clinical variables was explored using chi square comparisons or Student’s t and Wilcoxon tests according to the nature and distribution of the variables.

Multivariate logistic regression models were conducted to identify if patient characteristics or clinical variables were associated with recovery time of fatigue (Model 1), perceived stress using PSS (Model 2) and sleep quality (Model 3). Variables included in the model were chosen based on available literature, expert discussion and results of the bivariate analysis. A multivariate regression model was built for each e-PROMS, with the ePROMs as dependent variable, and clinical characteristics (age, gender, dialysis duration and cardiovascular history), undernutrition, hemoglobin, duration of hemodialysis session and number of sessions per-week, and the two other e-PROMs as independent variables. Undernutrition was defined by at least two of three following criteria: Serum Albumin < 35 g/l, Serum Prealbumin < 300 mg/l, nPCR < 1.2 g/Kg/d. Cardiovascular history was defined by at least one of the following: diabetes, coronary artery disease, heart failure and stroke. A bilateral threshold of 5% was considered to define the statistical significance. The analysis was performed with SAS 9.1 software.

### Ethical considerations

The study was declared to the data protection authority in France, known as the *Commission Nationale de l’Informatique et des Libertés* (CNIL), and was approved by the CPP Ile-de-France VII on 26 December 2019 in accordance with French regulations.

## Results

In total, 173 patients were included during the study period. The mean age of patients was 66.22 ± 14.4 years and majority of patients were males (68%). Patients were treated with hemodialysis for a mean total duration of 48.9 ± 58.02 months (median: 31 months) and were mainly treated in self-dialysis unit (67%). The mean length of dialysis per week was 11.46 ± 1.41 h corresponding to 3.09 ± 0.56 sessions per week (Table 3).

**Table 3:** Patient characteristics with chronic kidney disease (CKD) treated with hemodialysis in the ePROMs study

	Total	Center 1	Center 2	Center 3	*p*-value
Patients n (%)	173 (100)	72 (41.6)	44 (25.4)	57 (33)	
Age mean (±SD)	66.2 ± 14.4	71.8	58.7	64.5	< 0.001
Gender (Male) n(%)	117 (68)	50 (69)	31 (70)	36 (63)	0.674
Dialysis					
Duration in months mean (median)	48.9 (31)	35 (26.5)	62.9 (30)	56.9 (38)	0.107
Self dialysis unit (%)	67	44	61	100	< 0.001
Duration of session	11.5 ± 1.4	11.1 ± 1.5	12	11.6 ± 0.6	0.001
Number of sessions per week	3.1 ± 0.6	3 ± 0.5	3.2 ± 0.6	3.20.6	0.011
BMI n (%)					
< 18	10 (6)	4 (5,6)	4 (10)	2 (3,6)	0.01
18–25	73 (44)	22 (30,6)	22 (58)	29 (52,8)	
> 25	82 (50)	46 (64)	12 (32)	24 (43,6)	
Comorbidities %					
Undernutrition	16	21	22	5	0.029
Diabetes	28	43	16	16	< 0.001
Cerebrovascular disease	6	10	6	2	0.162
Coronary artery disease	34	48	18	28	0.004
Peripheral artery disease	22	32	5	19	0.005
Congestive heart failure	34	45	13	35	< 0.001
Cancer	28	46	24	11	< 0.001
Hemoglobin (g/dL)					
Mean (±SD)	11.26 ± 1.1	10.99 ± 1.33	11.60 ± 0.75	11.41 ± 0.95	< 0.001
< 10	14 (8)	13 (18)	0 (0)	1 (2)	
10 to 12	117 (70)	48 (67)	25 (66)	44 (77)	
> 12	36 (22)	11 (15)	13 (34)	12 (21)	
Albumin (g/dL)					
Mean (±SD)	39.6 ± 5.9	39.2 ± 3.8	39.7 ± 3.9	40.2 ± 2.9	0.004
≤35	21 (13)	14 (19)	6 (16)	1 (2)	0.009
> 35	146 (87)	58 (81)	32 (84)	56 (98)	
Single-pool Kt/V					
Mean (±SD)	1.44 ± 0.34	1.59 ± 0.37	1.09 ± 0.21	1.22 ± 0.25	< 0.001
≤1.2	48 (29)	12 (17)	3 (8)	33 (58)	< 0.001
> 1.2	118 (71)	60 (83)	34 (92)	24 (42)	

In terms of comorbidities, 73% of patients had at least one comorbidity with coronary artery disease (34%), congestive heart failure (34%), diabetes (28%), cancer (28%) and undernutrition (16%). Expected differences between centers were observed (shown in Table 3). Regarding laboratory test results, 70% of patients had hemoglobin (Hb) between 10 and 12 g/dL, and 8% had Hb inferior to 10 g/dL. Moreover, 13% of patients had their albumin inferior or equal to 35 g/dL, and 29% has single-pool Kt/V inferior or equal to 1.2.

The prevalence of fatigue was 72% [95% CI, 64.7 to 78.7%] with a mean severity score of 5.84 ± 2.12 on a zero to ten scale. Recovery time was more than 6 h for 25% [95% CI, 18.6 to 32%] of patients. 39% [95% CI, 32 to 47%] of patients have a stress level C (which meant that they did not know how to manage stress and perception of continuous threat which can negatively impact life and disease course). 27% [95% CI, 20 to 35%] of patients have a stress level B (they know to manage stress most of the time but it is not possible to manage stress in some situations). The average intensity score was 3.3 on a zero to ten scale.

Sleep quality was disrupted for 15% [95% CI, 9.6 to 20.6%] of patients. The self-perceived health status of patients was 6.2 ± 2.12 (on a zero to ten scale) and 78% [95% CI, 70.5 to 83.5%] of patients stated that they had not a worsened health status than the year before (Table 4). No statistical differences were observed between centers for the three e-PROMS.

**Table 4:** Patients with chronic kidney disease (CKD) treated with hemodialysis reported symptoms in the ePROMs study

	Total	Center 1	Center 2	Center 3
Fatigue n (%)	124 (72)	52 (72)	30 (70)	42 (74)
Fatigue intensity	5.8 ± 2.1	6 ± 2.4	5.8 ± 1.9	5.7 ± 1.9
Recovery delay (%)				
< 2 h	42	38	50	42
2 to 6 h	33	32	34	33
7 to 12 h	13	15	7	16
> 12 h	12	15	9	9
Health status	6.2 ± 2.1	6 ± 2.5	6.2 ± 1.8	6.5 ± 1.9
Health status compared to 1 year before				
Improved	40	40	39	41
More or less improved	9	11	9	5
Non changed	29	22	33	35
Altered	22	27	19	19
Stress intensity	3.7 ± 3	3.5 ± 3.2	3.3 ± 3	3.8 ± 2.9
Stress severity (%)				
A	34	29	36	37
B	27	29	25	26.32
C	39	42	39	37
Sleep quality (%)				
Improved	6	6	7	7
More or less good	20	18	18.5	23
Non changed	59	61	63	54
Altered	15	15	11.5	16

Results of the multivariate analysis are reported in Appendix 1. Fatigue recovery superior to 6 h was associated with the decreasing duration of HD sessions (OR 0.15; [95% CI, 0.04 to 0.58], *p* = 0.006) and with a higher stress level (OR 2.68; [95% CI 1.04 to 6.88], *p* = 0.041). Higher stress level was associated with female gender (OR 2.27; [95% CI 1.001 to 5.14], *p* = 0.05) and fatigue recovery superior to 6 h (OR 2.7; [95% CI, 1.05 to 6.92], p = 0.04). Sleep disturbance was associated with cardiovascular comorbidities (OR 5.08; [95% CI, 1.56 to 16.59], *p* = 0.007).

## Discussion

In this study, we identified a high prevalence of self-reported fatigue at 72% and important stress at 39% for CKD patients treated with hemodialysis. To our knowledge, this was the first study in France to assess ePROM questionnaires filled directly with a tablet by patients in a dialysis unit.

Fatigue was the most prevalent symptom identified in our study, in comparison to the other symptoms assessed. The prevalence of 72% was consistent with the range of previous published literature, which presented results from 60 to 97%. These results were additionally similar to the weighted mean prevalence of 71% estimated in a systematic review [18–20]. These results did not differ between centers even though patient characteristics and comorbidities differed.

Items collected from a patient were with a simple binary question and VAS instead of a dedicated measure and thus may not have reflected the specificity of fatigue from patients under hemodialysis. Such specific questionnaire was not available at the time of protocol definition and therefore a generic questionnaire was used. In future studies, items may be collected via the recently published measure SONG-HD questionnaire specifically designed for patients treated with hemodialysis [21]. This innovative tool designed through an international study included several components of fatigue including tiredness, lack of energy and inability to participate in social situations [22], however, did not distinguish between interdialytic fatigue and post-dialysis fatigue [23].

In our study, the post-dialysis fatigue through the after-session delay recovery time in hours was chosen to be assessed as expressed by the patient. The recovery time inferior to 6 h found in the study for 75% of patients were similar compared to an international study where 73% of patients declared the same timing, as well as a recovery time of more than 12 h declared by 12% of patients from our study compared to 10% in the international DOPPS study [24]. Increased fatigue and higher levels of perceived stress were associated in the multivariate analysis indicating the potential interrelation between these two symptoms.

The stress assessment through the perceived stress score seemed to be more informative than the VAS to assess its intensity as it allowed to better discriminate patient groups with different stress levels. The two scales are different as the VAS describe the stress at the time of the questionnaire while the PSS includes the stress felt in the past week. Additionally, the VAS may reflect the stress level more at the time of the questionnaire while the PSS findings may reflect the stress tendency over the past weeks more and thus may be a better estimation of the patient stress level at home. The high proportion of patients with a stress level C may indicate the need to provide patients with interventions to better manage their stress and improve clinical outcomes.

Since stress and fatigue were also related in the multivariate analysis, having a positive impact on one of the symptoms may also have a positive impact on the other. Further research to better understand the interrelations between fatigue and stress and characterize the profiles of patients that are more prone to experience high level of these symptoms may therefore be necessary. Furthermore, women were more prone to higher level of stress in our study. Therefore, gender could also affect dialysis outcomes and additional research is necessary to better tailor adapted educational interventions.

Results on sleep quality were different to those reported in the literature. While only 15% of patients with reported altered sleep were found, sleep disturbance was reported at weighted mean prevalence of 44% with a range of 20 to 83% [12, 25, 26] in various countries. The observed difference may be due to the questionnaire used to assess the sleep quality in our study. The questionnaire in our study focused on the sleep quality the night before the dialysis session in comparison to the previous nights, to assess the potential impact of pre-dialysis anxiety on sleep quality. Additionally, these results may be in favor of a limited impact of the dialysis on sleep quality. This difference with literature could be explained because the primary indicator has been the disturbance in overall sleep quality for patients treated by hemodialysis in comparison to their situation before the initiation of a dialysis [27]. Moreover, the relation identified in our findings between sleep disorders and cardiovascular comorbidities matches with the literature on interrelations between sleep and cardiovascular diseases [28, 29].

The main strengths of this study relied on the cross-sectional and multicenter design from various HD center settings and the use of simple questionnaires to collect data from patients through a tablet directly at patient side. The collection of data from patients during a consultation or HD session through a tablet device also allowed to not have missing data that could have weaken the interpretation of the results. On the other hand, the answers provided by patients may as well have been influenced by their location in the dialysis unit.

Despite significant differences in patient characteristics from the three centers including age, comorbidities or type of dialysis, no differences were found on the prevalence of the various PROMs, in favor of internally coherent results. The study population was not matching with the population profile of the French Renal Epidemiology and Information Network (REIN) [30–32] and consequently, in terms of comorbidities, coronary artery disease, congestive heart failure and cancer comorbidities the prevalence were higher in our study population. Contrarily, prevalence of diabetes and cerebrovascular disease were lower compared to REIN. This difference may lead to a selection bias and therefore our results may not be applicable to all hemodialysis patients in France. The 3 centers in our study have different characteristics, and no statistical differences were observed between centres for the three e-PROMS.

The main limitations of this study included the observational design, limited number of patients included, and the absence of linkage of PROMs with clinical outcomes such as cardiovascular events, hospitalizations or mortality. However, the objective was to first describe the symptoms of patients and not to explain these symptoms with their clinical situation or outcomes. It is also worth noting that this study did not assess whether better knowledge of patient reported symptoms could lead to better management of those symptoms by the care team, as it was noted in a recent study in Netherlands [33]. In terms of feasibility, time for completion and patient acceptability of the data collection process were also not collected.

Nonetheless, meta-analysis of oncology trials identified baseline fatigue as an independent prognostic factor for overall survival above performance status and quality of life in oncology patients, recommending collecting this information in routine oncology care for patient stratification [34]. Due to the clinical impact of fatigue on daily QoL of patients undergoing hemodialysis, it may be however relevant to consider the presence of reported fatigue in such patients to be a clinically relevant item to consider as itself, despite the need for further research in this area [35]. Additionally, recent studies identified an association between fatigue and all-cause mortality in those patients as well as between frailty and worse health related QoL [24, 36, 37].

To improve daily routine care of CKD patients treated with HD, the collection and integration of ePROMs into the care plan could be promoted in a standardized approach. Such efforts are currently being conducted in various countries or regions such as in Ontario, Canada with the Edmonton Symptom Assessment System Revised for routine PROMs collection in hemodialysis routine care and should be encouraged as well in France [38]. In this regard, the French Society of Nephrology, Dialysis and Transplant (SFNDT) published in 2020 a new guideline recommending the use of EuroQol 5D and 12-Item Short Form Health Survey for outcome measures and e-Satis national public system for measuring patient satisfaction [39, 40].

Dedicated software linking patient registries in hemodialysis, collection of ePROMs for remote patient monitoring and measures of patient satisfaction may thus be used to ease and improve routine care as well as clinical and epidemiological research [41].

## Supplementary Information

**Additional file 1.**

## Data Availability

The datasets used and/or analyzed during the current study are available from the corresponding author on reasonable request.
