# Supplementary material for: Patient-reported outcome measures in hemodialysis patients: results of the first multicenter cross-sectional ePROMs study in France
Source: BMC Nephrol. 2021 Oct 30;22:357. doi: 10.1186/s12882-021-02551-3 (PMC8556917; doi:10.1186/s12882-021-02551-3)
Supplement: Supplementary file 1 — Additional file 1. [file 12882_2021_2551_MOESM1_ESM.docx]

**Appendix 1**. Qualitative study interview guide, participant information and data of participant feedback

**Interview guide**

| **Step** | **Content** |
| --- | --- |
| **1** | Hello/good morning |
| **2** | Thank you for your time. I would like to know how long you have been treated with dialysis |
| **3** | How are your dialysis sessions generally going on? |
| **4** | Now I would like to discuss your feelings, experiences and your daily life... everything that can impact your daily life between two dialysis sessions. Do you understand?  I would like you to take a few minutes to go back in time to recall when you went back home after a dialysis session |
| **5** | How do you feel when you return back home after a dialysis session? |
| **6** | Can you tell me what symptoms (that you think are related to dialysis) are affecting your quality of life at home? |
| **7** | Can you tell me more about these symptoms? |
| **8** | If you had to rank them from most to least important, how would you rank them? |
| **9** | Have you discussed these .... symptoms with your dialysis centre's care team? |
| **10** | Is there anything else you would like to add? |
| **11** | Thank you for answering the questions |

| **Participant information** | | | | | | | |
| --- | --- | --- | --- | --- | --- | --- | --- |
| **Participant** | **Age** | **Gender** | **Marital status** | **Employment status** | **Time of interview** | **Type of haemodialysis** | **Approx. time on dialysis** |
| 01 | 64 | M | Married | Retired | During the dialysis session | Self-dialysis unit | 30 months |
| 02 | 77 | F | Married | Retired | During the dialysis session | In-centre | 144 months |
| 03 | 75 | M | Married | Retired | During a consultation | In-centre | 33 months |
| 04 | 77 | M | Married | Retired | During a consultation | In-centre | 71 months |
| 05 | 79 | M | Widowed | Retired | During a consultation | In-centre | 19 months |
| 06 | 87 | M | Married | Retired | During a consultation | In-centre | 102 months |
| 07 | 79 | M | Married | Retired | During a consultation | In-centre | 140 months |
| 08 | 64 | M | Married | Retired | During a consultation | Self-dialysis unit | 15 months |
| 09 | 55 | F | Divorced | Unemployed | During a consultation | Self-dialysis unit | 16 months |
| 10 | 74 | M | Married | Retired | During a consultation | Self-dialysis unit | 87 months |
| 11 | 70 | M | Married | Retired | During a consultation | Centre | 63 months |
| 12 | 80 | M | Married | Retired | During a consultation | Centre | 32 months |
| 13 | 83 | M | Widowed | Retired | During a consultation | Centre | 120 months |
| 14 | 72 | M | Married | Retired | During a consultation | Self-dialysis unit | 34 months |
| 15 | 66 | M | Married | Retired | During the dialysis session | Centre | 22 months |
| 16 | 88 | F | Widowed | Retired | During a consultation | Centre | 99 months |
| 17 | 70 | M | Married | Retired | During a consultation | Self-dialysis unit | 99 months |
| 18 | 60 | M | Married | Unemployed | During the dialysis session | Self-dialysis unit | 10 months |
| 19 | 81 | F | Widowed | Retired | Consultation | Centre | 144 months |
| 20 | 60 | F | Divorced | Unemployed | Consultation | Self-dialysis unit | 8 months |

**Data of participant feedback**

| Themes | Subthemes | Quotes |
| --- | --- | --- |
| **Fatigue** | Feeling tired | *"When I have to lose more than 4 kg, I know that the afternoon is going to be difficult for me.* (Interview 8)  *"I am exhausted after dialysis session, when I go home I have no energy anymore".* (Interview 18)  *"I have a dialysis session in the afternoon, I know that the dialysis at the beginning of the week, when I go home, I can't even eat, I sleep straight away, it's only the next day that I feel better*. (Interview 15) |
|  | Lack of energy | *"When I come back from dialysis, I can't do anything, I have to lie down all afternoon*". (Interview 2) |
| **Mental symptoms** | Feeling anxious | *"Here everyone is nice, but I cannot help it, I get tense as soon as I think about the dialysis machine.* (Interview 6) |
|  | Feeling irritable | *"When the dialysis goes badly, I go home, I do not talk to anyone, I stay in my room, I know that I can be very reactive for no reasons....* (Interview 9) |
|  | Feeling sad | *"I don't go to family meals anymore; it makes me sad not to share these moments with my children".* (Interview 1) |
|  | Feeling nervous | *"I weigh myself every day or even twice a day, as soon as I gain more than 3 kg in weight, it makes me anxious, because I have to restrict myself until the next dialysis and I know that the dialysis is likely to be difficult*". (Interview 10) |
|  | Concern | *"I am always worried, before I know who is going to punction the fistula, I have a difficult fistula, and most nurses don't get it right first time*". (Interview 17) |
| **Sleep disorders** | Difficulty falling asleep | *"The night before dialysis, I am already thinking about the next day's dialysis, who's going to inject me, how much weight I'll have to lose ... and I can't get to sleep*. (Interview 16) |
|  | Difficulty staying asleep | *"On the day of dialysis, I have to wake up at 5am, to be at the centre at 7am. I have to wash myself; my husband helps me.... the night before I think about all this so I wake up several times in the night".* (Interview 2) |
|  | Decreased sexual desire | *"Since the beginning of dialysis, I do not have erections anymore, I'm sure it's because of the dialysis, I didn't think it could happen to me at my age...* (Interview 18) |
|  | Dry mouth | *"When I come back from dialysis, my mouth is so pasty that my tongue sticks to the roof of my mouth...".* (Interview 3) |
|  | Cramps | *"When I get a muscle cramp at night, I have to get up and walk because it hurts so much...".* (Interview 19) |
|  | Itching | *"Itchy feeling doesn’t allow me to fall asleep ...".* (Interview 11) |

**Appendix 2**. Factors associated with fatigue recovery time more than 6 hours versus 6 hours or less, stress level measured using PSS B or C versus A, and sleep disturbance

| **Fatigue** | **OR** | **95%CI** | | **p-value** |
| --- | --- | --- | --- | --- |
| Age (per 5-year increase) | 0.98 | 0.84 to 1.14 | | 0.824 |
| Gender (female vs male) | 1.24 | 0.54 to 2.85 | | 0.619 |
| Dialysis vintage (per 1-year increase) | 0.98 | 0.89 to 1.08 | | 0.672 |
| Cardiovascular history* | 0.42 | 0.17 to 1.01 | | 0.054 |
| Undernutrition** | 1.28 | 0.42 to 3.93 | | 0.671 |
| Hemoglobin (per 1-g/dL increase) | 1.03 | 0.71 to 1.48 | | 0.894 |
| Duration of hemodialysis session (per 4 hours) | 0.15 | 0.04 to 0.58 | | **0.006** |
| Number of sessions per week | 0.92 | 0.39 to 2.13 | | 0.841 |
| PSS Stress (level BC versus A) | 2.68 | 1.04 to 6.88 | | **0.041** |
| Sleep disturbance | 1.94 | 0.64 to 5.65 | | 0.245 |
| **Stress** |  |  |  |  |
| Age (per 5-year increase) | 0.97 | 0.85 to 1.12 | | 0.686 |
| Gender (female vs male) | 2.27 | 1.001 to 5.14 | | **0.050** |
| Dialysis vintage. (per 1-year increase) | 1.01 | 0.94 to 1.08 | | 0.876 |
| Cardiovascular history* | 1.62 | 0.74 to 3.54 | | 0.231 |
| Undernutrition** | 0.88 | 0.33 to 2.34 | | 0.794 |
| Hemoglobin (per 1-g/dL increase) | 0.99 | 0.72 to 1.35 | | 0.923 |
| Duration of hemodialysis session (per 4 hours) | 1.78 | 0.54 to 5.82 | | 0.343 |
| Number of sessions per week | 1.03 | 0.53 to 2.03 | | 0.929 |
| Fatigue recovery (>6hrs versus 6hrs or less) | 2.70 | 1.05 to 6.92 | | **0.040** |
| Sleep disturbance | 0.67 | 0.25 to 1.82 | | 0.431 |
| **Sleep disturbance** |  |  |  |  |
| Age (per 5-year increase) | 0.84 | 0.69 to 1.02 | | 0.072 |
| Gender (female vs male) | 1.76 | 0.61 to 5.10 | | 0.297 |
| Dialysis vintage (per 1-year increase) | 0.93 | 0.81 to 1.07 | | 0.305 |
| Cardiovascular history* | 5.08 | 1.56 to 16.59 | | **0.007** |
| Undernutrition** | 0.38 | 0.08 to 1.91 | | 0.239 |
| Hemoglobin (per 1-g/dL increase) | 0.83 | 0.54 to 1.28 | | 0.394 |
| Duration of hemodialysis session (per 4 hours) | 1.47 | 0.26 to 8.42 | | 0.666 |
| Number of sessions per week | 0.86 | 0.28 to 2.58 | | 0.783 |
| Fatigue recovery (>6hrs versus 6hrs or less) | 0.67 | 0.24 to 1.86 | | 0.439 |
| Stress PSS (BC versus A) | 1.75 | 0.58 to 5.27 | | 0.317 |

OR; Odds Ratio, CI; confidence interval, PSS; perceived stress scale, * Cardiovascular history including at least one of the following: diabetes, coronary artery disease, heart failure and stroke. ** Undernutrition defined by at least two of three following criteria: Serum Albumin<35 g/l, Serum Prealbumin <300 mg/l, nPCR<1.2 g/Kg/d
